# A Complementary Sensory Tool for Children with Autism Spectrum Disorders

**DOI:** 10.3390/children7110244

**Published:** 2020-11-20

**Authors:** Sabina Barrios-Fernández, Margarita Gozalo, Beatriz Díaz-González, Andrés García-Gómez

**Affiliations:** 1 Medical-Surgical Therapeutics Department, University of Extremadura, 10003 Cáceres, Spain; 2 Psychology and Anthropology Department, University of Extremadura, 10003 Cáceres, Spain; mgozalo@unex.es; 3 Nursing and Occupational Therapy College, University of Extremadura, 10003 Cáceres, Spain; bdiazgon@alumnos.unex.es; 4 Education Sciences Department, University of Extremadura, 10003 Cáceres, Spain; agarcil9@unex.es

**Keywords:** sensory processing, emotional regulation, assessment, autism spectrum disorders

## Abstract

Background: Sensory integration (SI) issues are widely described in people with autism spectrum disorder (ASD), impacting in their daily life and occupations. To improve their quality of life and occupational performance, we need to improve clinical and educational evaluation and intervention processes. We aim to develop a tool for measuring SI issues for Spanish children and adolescents with ASD diagnosis, to be used as a complementary tool to complete the Rivière’s Autism Spectrum Inventory, a widely used instrument in Spanish speaking places to describe the severity of ASD symptoms, recently updated with a new sensory scale with three dimensions. Methods: 458 Spanish participants complemented the new questionnaire, initially formed by 73 items with a 1–5 Likert scale. Results: The instrument finally was composed of 41 items grouped in three factors: modulation disorders (13 items), discrimination disorders (13 items), and sensory-based motor disorders (15 items). The goodness-of-fit indices from factor analyses, reliability, and the analysis of the questionnaire’s classification capability offered good values. Conclusions: The new questionnaire shows good psychometric properties and seems to be a good complementary tool to complete new the sensory scale in the Rivière’s Autism Spectrum Inventory.

## 1. Introduction

### 1.1. Sensory Integration Process

Sensory integration (SI) is “the neurological process that organizes sensations from one’s own body (internal) and the environment (external) and makes it possible to use the body effectively within the environment” [1] (p. 11). An adequate organization of sensory information is necessary for producing adaptive responses in daily life, which includes different end products: motor, cognitive, behavioral, emotional, or learning outcomes [2]. SI is considered a prerequisite so that more complex functions, as perceptual-motor and cognitive ones, can be appropriately developed [3].

The SI process runs through a series of stages. Firstly, the sensory organs capture fragments of sensory information, which can have either an internal or an external origin. Later, that information is integrated in the central nervous system (CNS) to become a meaningful whole [4]. The SI process takes place in different brain structures in a coordinated way, classifying, and organizing the sensory flow through a series of stages. Firstly, in registration, the CNS detects the sensory sensations from our sensory receptors and we become aware of those sensations [5]. Next, in modulation, the CNS regulates and processes the sensory stimuli [6]. Then, during discrimination the CNS distinguishes between different sensory stimuli, perceiving their specific qualities and becoming meaningful [6,7,8]. Finally, we elicit a response, intended to adaptive, which can include attention, organization, self-esteem, self-confidence, movement, reasoning, and learning outcomes [1,7,9]. Within that end products, and in the group of adaptive motor-based responses, we must refer to praxis. Praxis is the ability to conceptualize, plan, and execute unusual motor actions. Thus, it allows us to organize and manage a purposeful interaction with the physical world, thus involving both motor and cognitive skills [8,10].

Although traditionally we have focused in five senses (vision, hearing, smell, taste, and touch), there are three more sensory systems essential to be successful in daily life: proprioception, vestibular system, and interoception. Proprioceptive sense reports on sensations from muscles, ligaments, and joints, providing information about the compression and stretching of muscles and joints. Proprioception and touch together form the somatosensory pathway, considered essential for praxis and movement [11,12]. The vestibular system provides information on movement, gravity and balance, so it is crucial for the building of spatial and temporal relationships [13]. It also provides information about the speed and the direction of the head movement and our position with relation to gravity [9]. Interoception sense processes sensory stimuli within the body, including body sensations (hunger, thirst, body temperature, heart, breathing rate, etc.) and emotional states (happiness, sadness, shame, anger), being intimately related to self-regulation and well-being [14,15]. 

### 1.2. Sensory Processing Disorders

When sensations flow in an organized and integrated way, our brain can use those sensations to form perceptions, behaviors, and learning; when the flow of sensations is disorganized, perception, behavior, and learning are like a traffic jam at a rush hour [16]. Therefore, when SI is not working properly, motor, cognitive, emotional, behavioral, and adaptive issues produce a decrease in daily living functioning and learning [17,18,19,20]. This dysfunction can be mild, medium, or severe [21]. Sensory processing disorder (SPD) is a neurological disorder in which the ability to process and interpret sensory stimuli results in abnormal responses, causing a decrease in the quality of life and occupational performance [22,23]. Several models have been developed to understand the SPD [1,6,24], being Miller’s model one of the most accepted. According to it, SPD can be classified into three categories with their corresponding subtypes: sensory modulation disorders, sensory discrimination disorders, and sensory-based motor disorders. Sensory modulation disorders happen when the CNS has problems in regulating the sensory information (degree, nature, or intensity) resulting in the following subtypes: sensory over-responsivity (exaggerated response), sensory under-responsivity (lack or insufficient response), or sensory craving (desperate seeking for sensory information). Sensory discrimination disorders happen when there is difficulty interpreting the qualities of the sensory stimuli. As a result, the responses are often slow, and sometimes, wrong. Finally, sensory-based motor disorders cause difficulty with motor planning and movement, resulting in postural disorder or dyspraxia subtypes [6,25].

### 1.3. Autism Spectrum Disorders and SPD Relationships

Taking the latest version of the Diagnostic and Statistical Manual of Mental Disorders (DSM-5) as a reference [26], ASD are included in the neurodevelopmental disorders group, and they are defined by the presence of (a) persistent deficits in social communication and interaction, and (b) restricted, repetitive patterns of behavior, interests, or activities. Within the (b) criterion and, for the first time in the DSM, sensory abnormalities were included as “Hyper- or hyporeactivity to sensory input or unusual interest in sensory aspects of the environment (e.g., apparent indifference to pain/temperature, adverse response to specific sounds or textures, excessive smelling or touching of objects, visual fascination with lights or movement)” (p. 50). 

With regards to etiology, and although it is widely recognized that genetic and environmental factors and their interactions contribute to the phenotypes of ASD, the precise causal mechanisms keep still unclear [27]. On a neuroanatomical basis, it is hypothesized that ASD symptoms should be a consequence of brain disconnection since hypomyelination of the brain nerves occurs simultaneously with the main behavioral symptoms [28]. Other studies complement this hypoconnectivity hypothesis by suggesting that in addition to hypoconnectivity in some regions of the cerebral cortex and at an interhemispheric level, a compensatory hyperconnectivity between the thalamus and the cerebral cortex, explaining sensory, and social symptoms [29]. Under this assumption, sensory issues in ASD have as origin atypical connectivity of neuronal structures. Nevertheless, it seems that topography of hypoconnectivity in ASD is unique and different from other conditions, such as SPD. In ASD, areas related to socio-emotional processing are highly affected; whereas, in SPD, there is lower connectivity in the brain’s perception and integration pathways, which serve as connections for the auditory, visual, and somatosensory systems involved in SI [30].

SI issues are commonly reported in ASD, compared to their peers [31]. Various studies have tried to explain the most frequent sensory profiles or those issues that cause the biggest issues in children with ASD, as well as the proposals of intervention to improve their occupational performance [19,31,32,33,34,35,36,37]. With regards to ASD specific sensory profiles, hyporeactivity/under-responsivity is one of the most consistent issues found [24], although hyperreactivity/over-responsivity and sensory seeking have been also reported [38]. Several studies have found relationships between the core symptoms of ASD and sensory impairments, such as repetitive behaviors [34,39], with social communication and interaction [31,40,41], but also, with movement issues, including coordination, planning, and timing [42,43], impacting in their daily life [44]. With regards to the interventions, some of the studies focused on Ayres’s Sensory Integration Therapy [36,45], and others in using specific sensory techniques and environmental modifications, thus the promotion of ecological approaches to improve occupational performance [4,37].

### 1.4. Autism Spectrum Disorders and SPD Assessment

There are different tools to measure SI functioning, including questionnaires, observational tools, and comprehensive tests administered to the children or adolescents. Some reviews have been performed to resume information about SI tools, noticing that there are a large number of proposals [46,47]. Other reviews have checked for the most used SI tools in ASD, providing information about their characteristics and limitations [48,49,50]. Some of the most representative instruments are the Sensory Profile (SP) [51] and its second version (SP2) [52], a group of standardized questionnaires for assessing sensory processing including the infant, toddler, child, short, and school companion forms, from birth to 14.11 years. The Sensory Processing Measure (SPM), formed by a set of questionnaires to assess SI in home and the school, in children between 5–12 years. It also includes self-evaluation forms to be completed by the children and adolescents. There is a preschool version from 2–5 years [53]. The Sensory Integration and Praxis Test (SIPT) is a comprehensive test formed by 17 subtests to assess visual, tactile, kinesthetic, and motor tasks in children from 4–8.11 years [16]. There are several emerging SI assessment tools. The Sensory Processing 3-Dimensions (SP3D) is a tool composed of a series of task to elicit typical and atypical behavioral responses in children, covering sensory modulation, discrimination, and sensory-based motor disorders; and by a questionnaire with five subscales: sensory over-responsiveness, sensory under-responsiveness, sensory craving, postural disorder, dyspraxia, and sensory discrimination disorder [25,54]. The Evaluation in Ayres Sensory Integration (EASI) is a comprehensive assessment test for SI which includes measures related to sensory perception, sensory responsiveness, postural, ocular and bilateral integration, and praxis [24,55]. 

**Figure 1:**
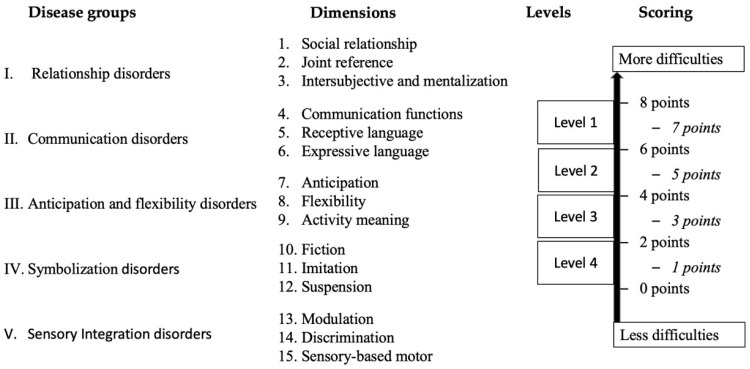
Summary of Rivière’s Autism Spectrum Inventory [60,61]. Disease groups from I–IV with their 12 dimensions correspond to Rivière’s original version. The V scale with the dimensions 13–15 was added by García-Gómez [62]. Preferred scores for rating the Inventory are the even ones, while odd scores are used to describe intermediate stages.

Tools for ASD assessment, including detection [56,57], diagnosis and measuring changes after interventions [58,59] are also available. In any case, the assessment of the severity of ASD should be complete and comprehensive and must include the measure of the SI and its impact in daily life. Within these tools, the Autism Spectrum Disorders Inventory developed by Rivière [60,61], is a widely used tool both in Spain and Latin America. It examines the severity of ASD by establishing four disease groups: relationship disorders, communication disorders, anticipation and flexibility, and symbolization, resulting in 12 dimensions, all of which can be scored from 0 to 8 points. The Rivière’s Autism Spectrum Inventory was set up before the importance of the SI was spread so, recently, a new sensory scale has been incorporated [62] updating the tool to the current knowledge of ASD. Now it is formed by five disease groups and 15 dimensions (Figure 1). An advantage of the Rivière’s Autism Spectrum Inventory is the fact that, as being designed by severity levels, it can help the clinicians’ in their judgment to determine the levels of severity required in DSM-5 [26]. However, and although this instrument explains the four levels of affectation in each dimension, it does not define specific behaviors to observe, so using complementary tools to collect information is strongly recommended. 

### 1.5. Aim

We aim to create a questionnaire to be used as a support for scoring the new sensory scale in the Rivière’s Autism Spectrum Inventory, a widely used tool to assess ASD severity in Spanish speaking places.

## 2. Methods

### 2.1. Participants

The sample was formed by 458 children and adolescents (308 males, 68.7%, and 144 females, 31.3%) from 4 to 19 years (*x* = 9.6, *dt* = 4.42). Of these, 259 were individuals with typical development (57.2%), 145 presented ASD clinical diagnosis (32%) and 54 had other diagnoses different than ASD resulting in intellectual, sensory, and/or motor disabilities (11.95%).

### 2.2. Procedure

After conducting a literature review, a group of experts in the fields of Occupational Therapy and Psychology, with clinical experience, created a preliminary version of the tool composed of 73 items. Then a pilot study was carried out with 31 ASD families with diagnosed children. The 50 items with the best indicators were selected. Participants were recruited using the snowball technique in the case of typical development children, and through different associations, in the case of diagnosed children. The data collection was carried out between May and August 2020. This protocol adheres to the updates of the Declaration of Helsinki, and the study was approved by the Committee on Biomedical Ethics of the University of Extremadura (97/2020).

Our instrument, the Behavioral Observation on Sensory Stimuli Questionnaire for Parents (BOSS-P) was administered to the families. They were also asked for socio-demographic data, including age, sex, clinical diagnosis, intellectual capacity, language level, comorbidities, and the need for aids in their daily life. Once the questionnaire was administered to the sample, the items were analyzed by the group of experts, discarding those which did not fit on the theoretical model, being the final version composed of 41 items (Supplementary Table S1). The BOSS-P was administered together with the Sensory Profile 2 (SP2) Short Form [63,64] to 31 participants, to obtain validity indicators.

### 2.3. Instrument

The BOSS-P, a new instrument to better characterize ASD children and adolescents to fulfil the three new sensory dimensions from Rivière’s Autism Spectrum Inventory based on Miller’s model, must be completed interviewing with main caregivers, which may answer the 41 items through a Likert scale with five response options, from 1 to 5 (higher scores mean greater SI dysfunction). It takes about 25–30 min to complete the interview.

### 2.4. Statistics

To perform the validation process of the BOSS-P we have carried out: (1) an exploratory factor analysis (EFA), (2) confirmatory factor analysis (CFA), (3) reliability analysis, (4) the assessment of concurrent validity through the correlations with the SP2, and (5) provide descriptive statistics from the typical development and the ASD subsamples.

Because we are handling ordinal variables from a Likert-type scale with five response categories, the EFA was carried out with the FACTOR software [65,66,67] using polychoric correlations and robust methods [68]. Items with factorial weights below 0.30 were excluded. The CFA was carried out with the IBM SPSS AMOS^TM^ 24 [69] using the Maximum Likelihood estimation procedure, suitable for Likert-type scales of five response categories. The CFA supports the factorial solution provided by the EFA and also offers the model of relations between the variables that best fits with the data [70,71,72].

The evaluation of the model fit was made taking into account the Chi-Square divided by degrees of freedom (CMIN/DF) and the p of Chi-square following Byrne’s criteria [73]. The statistical *p* of Chi-square is dependent on the sample size, so it was convenient to use other goodness-of-fit indicators choosing the Tucker–Lewis index (TLI), the comparative fit index (CFI) following Hu and Bentler’s criteria [74], the root-mean-square error of approximation (RMSEA), and the root-mean-square residuals (RMSR) [75,76]. 

Ordinal alpha coefficients were calculated [77,78] to assess reliability, considering values >0.70 acceptable and >0.90, excellent [79]. The analysis of the correlations between our tool and the SP2, the descriptive statistics of the subsamples and the relative operating characteristic (ROC) analysis were carried out to check the instrument’s ability to classify between the two subsamples, using the IBM SPSS^TM^ 24 [80] statistical package. Cohen’s d statistic [81] was also calculated to check the magnitude of the effect size of the differences between the subsamples scores.

## 3. Results

### 3.1. Exploratory Factor Analysis

After administering the experimental version of the questionnaire to the sample, a solution of 41 items grouped into three correlated factors was obtained. Bartlett’s (5025.4; *df* = 820; *p* = 0.000) and Kaiser–Meyer–Olkin test (0.912) statistics showed a very good sample suitability [82]. In Table 1, can be found both the rotated factorial matrix and factorial weights of each item. The three factors obtained represent (F1) modulation disorders with 13 items, (F2) discrimination disorders with 13 items, and (F3) sensory-based motor disorders with 15 items.

With regards to the correlation between factors, moderate relationships were found between F1–F2 (0.38); F1–F3 (0.61), and F2–F3 (0.53) [81], which was to be expected since they are different stages within the same neurobiological process.

### 3.2. Confirmatory Factor Analysis

**Figure 2:**
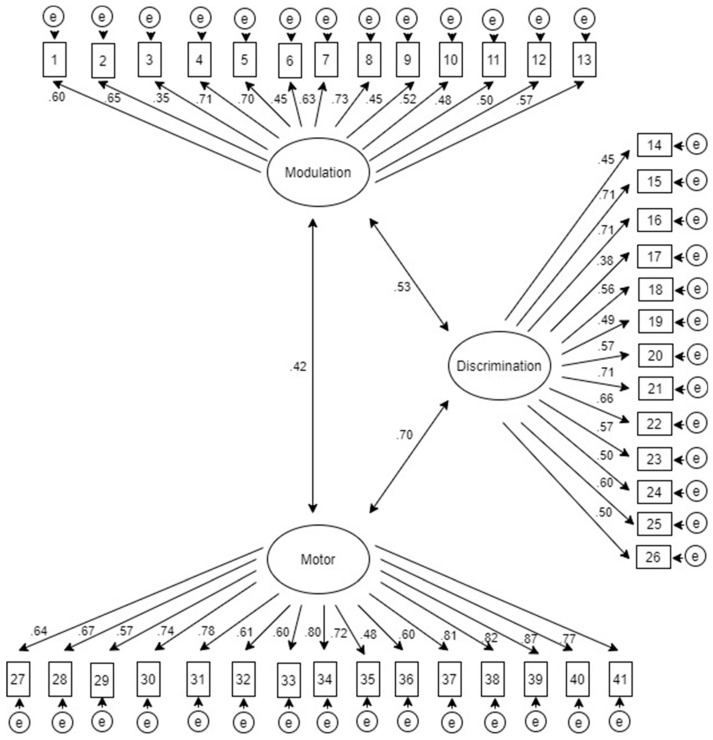
The Behavioral Observation on Sensory Stimuli Questionnaire for Parents’ (BOSS-P) graphical representation after confirmatory factor analysis (CFA).

The CFA confirms the exploratory factorial solution revealing three latent variables which group the 41 observable variables (items). Figure 2 shows the graphical representation of the analyzed model, being (F1) modulation disorders, (F2) discrimination disorders, and (F3) sensory-based motor disorders. The factorial weights of every item and the covariation relations between the latent variables are shown.

In Table 2, are represented the goodness-of-fit indices from the CFA, showing good values.

### 3.3. Reliability

To analyze the concurrent validity, we compared the BOSS-P with the SP2, a tool for SI assessment validated for Spanish children and adolescents. Both questionnaires were administered to 31 participants with ASD to study their correlations. As shown in Table 3, the modulation disorders factor (F1) from the BOSS-P was the only with significant and moderate correlations with the factors analyzed in the SP2.

### 3.4. Questionnaire’s Capability to Classify between ASD and Typical Development 

Descriptive statistics of participants with ASD (*n* = 145) and with typical development (*n* = 259) subsamples are shown in Table 4. It can be checked that both, mean and standard deviation of every subsample offer different scores.

**Figure 3:**
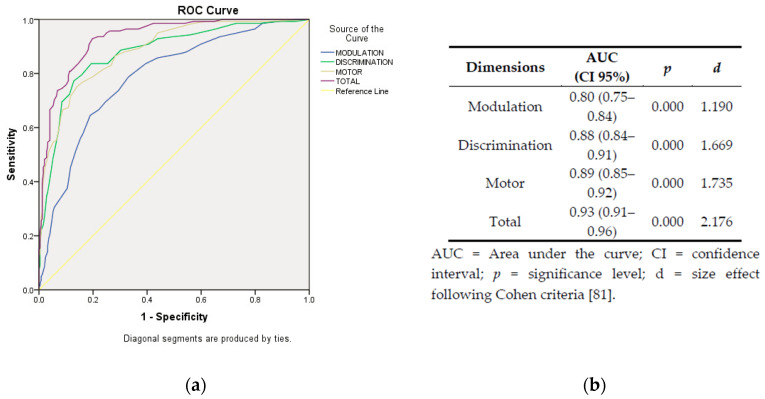
Graphical representation (**a**) and statistics (**b**) from the receiver operating characteristic (ROC) curves.

In Figure 3, graphical representation and statistics from ROC curves are provided. The area under the curve (AUC) shows differences with large effect magnitudes between the three factors, being the BOSS-P total score the most capable dimension to establish a correct classification of subjects according to their reference group.

Considering the Rivière’s Autism Spectrum Inventory scoring system, an approximation to the level of SI severity using the level of affectation in the Rivière’s inventory and the BOSS-P interquartile scores was obtained in the ASD sample (see Table 5).

## 4. Discussion

### 4.1. About the BOSS-P Questionnaire

We aimed to create a questionnaire to support the new SI scale [62] added to the Rivière’s Autism Spectrum Inventory [60,61]. The Rivière’s Inventory is a widely used instrument in Spain and Latin America, which allows us to establish the level of the ASD severity, in line with the levels proposed in the DSM-5 [26]. The Rivière’s Inventory is useful both during the diagnosis and intervention processes.

The BOSS-P is a screening instrument, administered through an interview with parents or carers, which is not intended to replace other comprehensive assessments, existing or emerging, with good psychometric properties on SI. However, our instrument has several advantages: (1) it is a quick test which is administered in 25–30 min; (2) that does not require specific training; (3) is open access; (4) is a complete tool, as it assesses items within the three areas described by Miller [6]: sensory modulation, sensory discrimination, and sensory-based motor disorders; (5) with good psychometric properties in terms of validity, reliability, and discrimination capacity; (6) created in Spain and therefore, adapted to the cultural characteristics of this country; (7) which fills a gap in terms of SI tools in Spanish-speaking population; and (8) which complements a psychological test widely used in the Spanish-speaking world, the Rivière’s Inventory for people with ASD.

We have also provided an attempt to the combined use of the BOSS-P and the Rivière’s Inventory, by linking the Rivière’s level of severity and the BOSS-P quartile scores. Our instrument showed good psychometric values, offering a factorial structure formed by the three groups proposed by Miller [6]. These data are interesting because let us verify that Miller’s model is consistent in different cultures and because. The BOSS-P’s ability to classify between participants with ASD and typical development children seems adequate (AUC = 0.938), corresponding to a large effect size between the scores of both subsamples (*d* = 2.176) [83]. 

### 4.2. The BOSS-P and Other Instruments

Some reviews have found that psychometric properties of some of the SI tools are from poor to moderate, so the professionals must use the obtained data with caution [48,50], selecting appropriate SI assessments depending on the detected SI needs [47]. However, as aforementioned, there are few available instruments for Spanish children and adolescents. According to our best knowledge, neither the SIPT—considered as a Gold Standard for SI assessment [84]—nor the SPM are available for Spanish population, while the SP3D and the EASI are not yet published, being the SP2 the only tool of choice in Spain. The SP2 Spanish version covers a little shorter age range than the original, from 3 to 14.11 years. The BOSS-P covers from 4 to 19 years, a wider range including the full adolescent stage. The correlations between the BOSS-P and the SP2 only find relationships in modulation disorders, which could lead us to consider the necessity of using different tools to obtain information about SI if using the SP2. 

Concerning its psychometric properties, the BOSS-P items present excellent internal consistency (alpha > 0.87), similar or superior other questionnaires used in the international context [46]. The ability of the questionnaire to discriminate between sub-samples offers a large effect size (*d* = 2.176), which is slightly higher than the size effect of the difference reported in other instruments [85]. 

### 4.3. Limitations and Future Lines

This research has some limitations. The information was completed through parents, and although instruments completed by families are considered to be valid [86], we must be careful because some parents should overestimate or underestimate the development of their children [87]. The sample was one of convenience. Another limitation was that we could not perform a test–retest. As future lines, we will try to improve the psychometric properties of the questionnaire, as well as to perform studies for its use in other Spanish-speaking countries different than Spain.

## 5. Conclusions

The preliminary study of the psychometric properties of Behavioral Observation on Sensory Stimuli Questionnaire for Parents (BOSS-P) shows good values for its use in Spanish children and adolescents diagnosed with ASD between 4 and 19 years. This tool was designed to help clinicians and educational professionals to establish the level of severity in children and adolescents with ASD diagnosis through the new SI scale in the Rivière’s Autism Spectrum Inventory.

## Figures and Tables

**Table 1:** Rotated factorial matrix and factorial weights of each item.

Items	F1	F2	F3
1. Shows disproportionate reactions if touched.	0.491		
2. Shows panic reactions to loud noises.	0.624		
3. Shows rejection of water when showering or washing.	0.340		
4. He is bothered by noisy and crowded places.	0.829		
5. When something goes wrong, it takes a long time to calm down.	0.566		
6. Shows discomfort with activities that involve spinning.	0.507		
7. Cannot concentrate or perform tasks when background noise.	0.627		
8. He gets agitated in the presence of very powerful light sources.	0.760		
9. Frequently touches or puts body parts or objects in his mouth.	0.394		
10. He is bothered with strong smells.	0.702		
11. Some clothes bother him; he feels itchy about some fabrics.	0.730		
12. He dislikes personal hygiene or grooming activities.	0.452		
13. Quick movements are unpleasant for him.	0.643		
14. Attends to his name or when he is called.		0.492	
15. Communicates feelings aimed at satisfying basic needs.		0.620	
16. Realizes when he is tired or exhausted.		0.639	
17. Shows comfort when hugged by parents or close relatives.		0.837	
18. Shows satisfaction when basic needs are met		0.959	
19. When he is disconsolate, he gets calmed by his parents.		0.720	
20. Expresses enjoyment or feels comfortable in certain situations.		0.897	
21. Can perceive danger in situations that could harm.		0.475	
22. Can identify basic emotions in himself and others.		0.442	
23. Can orientate himself in the environment.		0.418	
24. Notices that his heart is racing when he is tired or excited.		0.522	
25. Recognizes the elements that make him nervous.		0.578	
26. Has difficulty in recognizing people’s faces.		0.374	
27. Has difficulty identifying parts of his own body.			0.655
28. Presents inability to reproduce speech movements.			0.737
29. Can ride a bicycle, rollerblades or a skateboard.			0.623
30. Can perform simple motor imitations.			0.724
31. Can fasten buttons or make loops to get dressed.			0.927
32. Can stack small blocks or string beads on a string.			0.569
33. Can use cutlery with both hands.			0.634
34. Can make copies from simple drawings.			0.930
35. Shows clumsiness in typing or using the computer keyboard.			0.814
36. Shows insecurity going downstairs/hills, holds on to railings.			0.485
37. Can adjust his strength when grasping objects.			0.452
38. Can cut with scissors properly for his age.			0.929
39. Can draw or colour within the proposed margins.			0.924
40. Can follow motor imitations containing multiple steps.			0.892
41. Can complete drawings with one half of it missing.			0.930

(F1) Modulation disorders; (F2) discrimination disorders; and (F3) sensory-based motor disorders. Items translated for readability; no cross-cultural adaptation performed.

**Table 2:** BOSS-P goodness-of-fit indices from the confirmatory factor analysis (CFA).

Indices	Cut-Off	Value
CMIN/DF	<2	1.995
*p* (χ^2^)	>0.05	0.000
TLI	>0.90	0.912
CFI	>0.90	0.925
RMSEA	<0.06	0.047 (0.043–0.051)
RMSR	<0.08	0.071

*p* (χ^2^): chi-squared probability; CFI: comparative fit index; NNFI: non-normed fit index, RMSEA: root mean square error of approximation; RMSR: root mean square of residuals.

**Table 3:** Correlation matrix between the Behavioral Observation on Sensory Stimuli Questionnaire for Parents (BOSS-P) and the Short Sensory Profile 2 (SP2).

	BOSS-P				SP2		
	F1	F2	F3	Total	Sensory	Behavioral	Total
F1	1						
F2	−0.076	1					
F3	−0.134	0.297	1				
Total	0.438 *	0.636 **	0.701 **	1			
Sensory	0.448 *	0.084	0.027	0.309	1		
Behaviour	0.600 **	0.034	0.147	0.446 *	0.613 **	1	
total	0.590 **	0.063	0.103	0.426 *	0.879 **	0.915 **	1

*. Correlation is significant at the 0.05 level (2-tailed). **. Correlation is significant at the 0.01 level (2-tailed) F1 = Modulation Disorders factor; F2 = Discrimination Disorders factor; F3 = Sensory-Based Motor Disorders factor; Sensory = Sensory Processing; Behavioral = Behavioral Responses associated with Sensory Processing.

**Table 4:** Descriptive statistics of ASD and typical development samples.

	Autism Spectrum Disorder				Typical Development			
	F1	F2	F3	Total	F1	F2	F3	Total
x	33.8	31.9	40.3	106	24.8	20.5	23.1	68.4
SD	8.9	8.0	11.9	19	7.7	5.5	7.8	16.0

x: mean; SD: standard deviation.

**Table 5:** Combination of the level of affectation in the Rivière’s Inventory and the Behavioral Observation on Sensory Stimuli Questionnaire for Parents’ (BOSS-P) interquartile scores.

			BOSS-P	
		F1	F2	F3
Rivière’s inventory levels of severity	1 (8 points)	>40	>36	>50
	2 (6 points)	34–40	30–36	40–50
	3 (4 points)	27–34	27–30	31.5–40
	4 (2 points)	<27	<27	<31.5

F1 = modulation disorders factor; F2 = discrimination disorders factor; F3 = sensory-based motor disorders factor.

## Supplementary Materials

The following are available online at https://www.mdpi.com/2227-9067/7/11/244/s1, Table S1: Behavioral Observation on Sensory Stimuli Questionnaire for Parents (BOSS-P) Versión original española: Cuestionario de Observación de la Conducta ante Estímulos Sensoriales para Padres de niños/as y adolescentes con Autismo/TEA (OCS-P).
