# Supplementary material for: A Complementary Sensory Tool for Children with Autism Spectrum Disorders
Source: Children (Basel). 2020 Nov 20;7(11):244. doi: 10.3390/children7110244 (PMC7699787; doi:10.3390/children7110244)
Supplement: Supplementary file 1 [file children-07-00244-s001.pdf]

Table S1. Behavioral Observation on Sensory Stimuli Questionnaire for Parents (BOSS-P). Versión original española: Cuestionario de Observación de la Conducta ante Estímulos Sensoriales para Padres de niños/as y adolescentes con Autismo/TEA (OCS-P).

	**TRASTORNOS DE LA MODULACIÓN SENSORIAL**
**1.**	Muestra reacciones desproporcionadas cuando se le toca
2.	Muestra reacciones de pánico ante ruidos fuertes
3.	Muestra reacciones de rechazo al agua a la hora de ducharse o lavarse
4.	Le molestan los lugares ruidosos y con mucha gente
5.	Cuando algo le contraría tarda mucho en recuperar la calma
6.	Muestra desagrado ante actividades que impliquen giros o volteretas
7.	No puede concentrarse o realizar tareas cuando hay ruido de fondo
8.	Se pone nervioso ante fuentes luminosas muy potentes
9.	Toca o se lleva a la boca partes del cuerpo u objetos de forma frecuente
10.	Le molestan especialmente los olores fuertes
11.	Le molestan algunas prendas de vestir, siente picor por algunos tejidos.
12.	Le desagradan las actividades de higiene o mantenimiento personal
13.	Los movimientos rápidos le resultan desagradables
	**TRASTORNOS DE LA DISCRIMINACIÓN SENSORIAL**
14.	Atiende a su nombre o cuando se le llama
15.	Comunica sensaciones destinadas a satisfacer necesidades básicas
16.	Se da cuenta de cuando está cansado o agotado
17.	Muestra agrado cuando es abrazado por padres o familiares cercanos
18.	Muestra placer cuando se satisfacen necesidades básicas
19.	Cuando está desconsolado se calma por las atenciones de los padres
20.	Manifiesta placer o se sentirse a gusto ante determinadas situaciones.
21.	Es capaz de percibir el peligro ante situaciones que puedan generar daños
22.	Es capaz de identificar en sí mismo y en los demás, emociones básicas
23.	Es capaz de orientarse en el espacio
24.	Nota que tiene el corazón acelerado cuando está cansado o excitado
25.	Reconoce las cosas que le ponen nervioso
26.	Tiene dificultades para el reconocimiento de las caras de las personas
	**TRASTORNOS MOTORES DE BASE SENSORIAL**
27.	Presenta dificultades para identificar las partes de su propio cuerpo
28.	Presenta incapacidad para reproducir movimientos del habla
29.	Es capaz de montar en bicicleta, en patines o en patinetes
30.	Es capaz de realizar imitaciones motoras simples
31.	Es capaz de abrocharse los botones o hacer lazadas para vestirse
32.	Es capaz de apilar bloques pequeños o de ensartar cuentas en una cuerda
33.	Es capaz de utilizar cubiertos con ambas manos
34.	Es capaz de realizar copias de dibujos simples.
35.	Muestra torpeza para escribir, utilizar el teclado del ordenador…
36.	Muestra inseguridad bajando escaleras o cuestas y se agarra a barandillas
37.	Es capaz de regular la fuerza a la hora de agarrar los objetos
38.	Es capaz de recortar con tijeras adecuadamente para su edad
39.	Es capaz de dibujar o colorear dentro de los márgenes propuestos
40.	Es capaz de realizar imitaciones motoras que contienen varios pasos
41.	Es capaz de completar dibujos a los que le falta la mitad
